# Secure tumor classification by shallow neural network using homomorphic encryption

**DOI:** 10.1186/s12864-022-08469-w

**Published:** 2022-04-09

**Authors:** Seungwan Hong, Jai Hyun Park, Wonhee Cho, Hyeongmin Choe, Jung Hee Cheon

**Affiliations:** 1 Department of Mathematical Sciences, Seoul National University, 1, Gwanak-ro, Gwanak-gu, Seoul, Republic of Korea; 2 Cryptolab Inc., 1, Gwanak-ro, Gwanak-gu, Seoul, Republic of Korea

**Keywords:** Homomorphic encryption, Multi-label classification, Privacy, Neural network, Softmax activation

## Abstract

**Background:**

Disclosure of patients’ genetic information in the process of applying machine learning techniques for tumor classification hinders the privacy of personal information. Homomorphic Encryption (HE), which supports operations between encrypted data, can be used as one of the tools to perform such computation without information leakage, but it brings great challenges for directly applying general machine learning algorithms due to the limitations of operations supported by HE. In particular, non-polynomial activation functions, including softmax functions, are difficult to implement with HE and require a suitable approximation method to minimize the loss of accuracy. In the secure genome analysis competition called iDASH 2020, it is presented as a competition task that a multi-label tumor classification method that predicts the class of samples based on genetic information using HE.

**Methods:**

We develop a secure multi-label tumor classification method using HE to ensure privacy during all the computations of the model inference process. Our solution is based on a 1-layer neural network with the softmax activation function model and uses the approximate HE scheme. We present an approximation method that enables softmax activation in the model using HE and a technique for efficiently encoding data to reduce computational costs. In addition, we propose a HE-friendly data filtering method to reduce the size of large-scale genetic data.

**Results:**

We aim to analyze the dataset from The Cancer Genome Atlas (TCGA) dataset, which consists of 3,622 samples from 11 types of cancers, genetic features from 25,128 genes. Our preprocessing method reduces the number of genes to 4,096 or less and achieves a microAUC value of 0.9882 (85% accuracy) with a 1-layer shallow neural network. Using our model, we successfully compute the tumor classification inference steps on the encrypted test data in 3.75 minutes. As a result of exceptionally high microAUC values, our solution was awarded co-first place in iDASH 2020 Track 1: “Secure multi-label Tumor classification using Homomorphic Encryption”.

**Conclusions:**

Our solution is the first result of implementing a neural network model with softmax activation using HE. Also, HE optimization methods presented in this work enable machine learning implementation using HE or other challenging HE applications.

## Background

Cancer is a disease caused by the unlimited proliferation of certain cells in the human body, and the exact cause of cancer is not yet known. In 2020 alone, 19.3 million new cases were reported worldwide, and 10 million people died because of cancer [1]. For this reason, the prediction of cancer through genetic data analysis has been regarded as one of the most important tasks since early treatment can reduce the lethal effects of cancer on the human body.

Machine learning (ML) is one of the fields of artificial intelligence that learns the process of finding solutions on its own without human assistance to a given problem. Due to the difficulty in the process of diagnosing tumors through genes, tumor classification using ML-based on large amounts of genetic data contributes to decision making to diagnose and treat cancer. Some cancer genome studies using ML techniques on large-scale data have shown the relationship between genetic modification and specific cancer types [2–5].

Since genetic data contains a lot of personal information and cannot be discarded or changed even if it is leaked, it is essential to protect the privacy of information about genetic data in the data analysis using ML. Various techniques, including differential privacy [6, 7] or multi-party computation [8], have been used to ensure privacy in the data analysis process. However, each of these approaches has the disadvantage of losing accuracy in the process of anonymizing the data or requiring multiple phases in the process of data sharing.

Homomorphic Encryption (HE) is a cryptographic scheme that enables us to perform arithmetic operations between encrypted data without decryption. HE has been considered one of the useful applications for privacy-preserving ML, as it allows the computation of desired operations without disclosing information about the data [9, 10]. However, most HE libraries [11–14] mainly support only addition and multiplication of arithmetic operations. Although linear operation in ML, such as matrix-vector multiplication, can be easily computed by using a suitable data packing method [15], many activation functions widely used in neural networks, such as sigmoid, ReLU, or softmax functions, cannot be directly operated for encrypted data. Most of the applications using HE overcomes such problems by approximating non-arithmetic operations with a polynomial that minimizes the error in a certain interval. The main problem with applying HE is to minimize computational increases occurring during the polynomial approximation process while limiting errors.

Integrating Data for Analysis, Anonymization and SHaring (iDASH) has held an annual secure genome analysis competition since 2014. Each year, important topics in the field of genetic analysis are selected to compete for the most effective solution. The problem of multi-label tumor classification using HE was one of the three tasks for the 2020 iDASH competition. Given the dataset with a total of 2,713 patients and their 25,128 genes, participants had to preprocess the given data, train the ML model with plain data, and obtain the highest microAUC score [16] within 5 minutes when the inference step was performed with encrypted test data of 909 patients.

### Related works

Integrated analysis, which means integrating and classifying different types of data for samples in the same cohort, was developed with the emergence of ML techniques. Now various ML techniques are used in multi-level omics data integration as reviewed in [17–22] and classified depending on the learning method, data integration method, and feature selection method: supervised and unsupervised learning; horizontal and vertical data integration; supervised and unsupervised feature selection.

In the field of cancer type classification based on somatic mutations, many ML techniques are used to build suitable multi-label classifiers. Classifiers using unsupervised learning method such as cluster analysis exists [23]; however, supervised learning-based classifiers are more in our interest because accuracy can be increased substantially with the labeled data. Chen et al. [24] used a supervised learning technique named Support Vector Machine (SVM) to classify cancer types of given somatic mutation samples.

Yuan et al. [4, 25] proposed DeepGene and DeepCNA, which are multi-label cancer classifiers based on Deep Neural Networks (DNN) and Convolutional Neural Networks (CNN), respectively. In particular, DeepGene uses a feature selection technique called Clustered Gene Filtering (CGF) based on cluster analysis. Sun et al. [26] also used DNN model with 5 layers and reached 70.1% classification accuracy. Some classifiers use multiple ML techniques and ensemble them to reach higher accuracy. Lee et al. [27] introduced a classifier named CPEM, an ensemble of two ML techniques, Random Forest (RF) and DNN, and reached 84.7% accuracy. However, these previous classification techniques do not consider privacy protection and sensitive genetic data leaks to the untrusted classifier owner, unless the classifier owner gives his whole model to the client.

As a solution of privacy-preserving ML for genetic data, ML over encrypted data with HE is drawing attention with the annual iDASH competition [9, 10, 28–35]. Kim et al. introduced a privacy-preserving logistic regression model over HE [28]. The model uses modified Nesterov’s accelerated gradient descent method to reduce the number of iterations, since the depth of the homomorphic circuit highly affects to the computational cost. The classifier was selected as the best solution of Track 3 at iDASH competition 2017, where similar approaches were also proposed [9, 29, 30].

Some parallelized versions submitted for the second track of iDASH 2018 competition [10, 31–34]. In 2019, the HE task of iDASH competition was a secure genotype imputation using HE. The solution showed “ultra-fast” HE models to verify genotype imputation that took less than 10 seconds for evaluation with only a 2–3% decrease in accuracy [35]. The authors also have confirmed that similar results can be obtained for various HE libraries, including BFV [36], CKKS, and TFHE [37].

### Difficulty of ML using HE

Some limitations of computation using HE make the general ML techniques not directly applicable to the ML over HE since they are yet insecure nor impractical for some reasons. First, as mentioned above, most HE libraries [11–14] mainly support only addition and multiplication of arithmetic operations. Thus, HE requires a polynomial approximation of non-polynomial functions such as ReLU, Sigmoid, softmax, and even division and comparison and those greatly amplify the amount of computation over HE. However, the supervised ML models [4, 25–27] generally have large circuit depths for high accuracy and use non-polynomial functions such as ReLU or max-pooling, which have high time complexity when implemented with HE.

To overcome the above problems, the ML over HE is being studied in various ways, such as replacing a non-polynomial such as sigmoid in logistic regression with a simple polynomial [28], or finding an algorithm consisting of only polynomial operations although it is less efficient than state-of-the-art algorithms involving non-polynomials [9]. In our case, we propose the efficient polynomial approximation of softmax function and our algorithm with HE that operates within a reasonable time.

Secondly, preprocessing methods included in the classifiers can leak personal information. To prevent leakage, preprocessing should be done by the client herself or the server in an encrypted state. However, clients with low computational capabilities cannot follow heavy preprocessing methods [24, 27]. Also in the case of servers, they should compute only with the data encrypted with HE, so the preprocessing will be very impractical due to logical/non-polynomial functions in the preprocessing as in [4, 24–27] when using the CKKS [14] HE scheme. Therefore, the classifiers with HE-friendly models and light and secure preprocessing are important for privacy-preserving classification.

In addition, if preprocessing method can be easily done by the server using above properties, there is another advantage to the client that does not have to compute preprocessing. In this case, the data can be used for various training models and so the client only needs to encrypts the original data and share them.

Table 1 summarizes the previous multi-label tumor type classifications and their weaknesses when applied to the privacy-preserving classification scenario. *HE-friendly Model* indicates the applicability of the model over HE, and *Client* and *Server (HE)* indicate the hardness of secure preprocessing as mentioned above.

**Table 1:** Multi-label classification of tumor type based on somatic mutation data. In the dataset, the numbers in parentheses are the number of tumor types used in classifications. The hardness of the client-side preprocessing is mainly due to high memory use, and the server-side preprocessing relies on the CKKS scheme

Reference	Dataset (Class)	Preprocessing in Train	Model	Acc	Preprocessing in Inference		HE-friendly
					Client	Server (HE)	Model
Chen et al. [24]	COSMIC (17)	filter & match to	SVM	62%	Hard	Hard	Yes
		KEGG pathways					
Yuan et al. [4]	TCGA (12)	filter (CGF) &	DNN	65.5%	Easy	Hard	No
		sparsity reduction (ISR)	(4 layers)				
Yuan et al. [25]	COSMIC (25)	regularize (clip into [0,10]) &	CNN	57.4%	Easy	Hard	No
		reshape (1D ⇔ 2D image)	(7 layers)				
Sun et al. [26]	TCGA (12)	filter & reference	DNN	70.1%	Easy	Hard	No
	1000GP (healthy)	with healthy	(4 layers)				
Lee et el. [27]	COSMIC (31, 12)	filter & various	RF + DNN	84.1%	Hard	Hard	No
		feature constructions	(3 layers)	(84.7%)			
Ours	TCGA (11)	filter	SNN	85%	Easy	Easy	Yes
			(1 layer)				

### Scenario and security model

The scenario in this paper follows the model presented in the HE task of iDASH 2020, which is also commonly considered in standard HE application models. In our scenario, two parties are involved in: the private data owner and the service provider, and they are simply denoted by client and server, respectively. The server trains the model using his data(or public large-scale data) and wants to provide a service to generate inference results, the prediction of the tumor type, from the client’s encrypted data. The client has limited computational power and wants to outsource the tumor type prediction; however, she does not want to leak her sensitive data. In addition, the client’s data needs to be preprocessed to fit the model computation while maintaining privacy in two possible ways: by the client with limited computation power or by the server in the encrypted state. Therefore, in our scenario, the server should not be aware of the client’s data when preprocessing as well as computing the inference results.

**Fig. 1:**
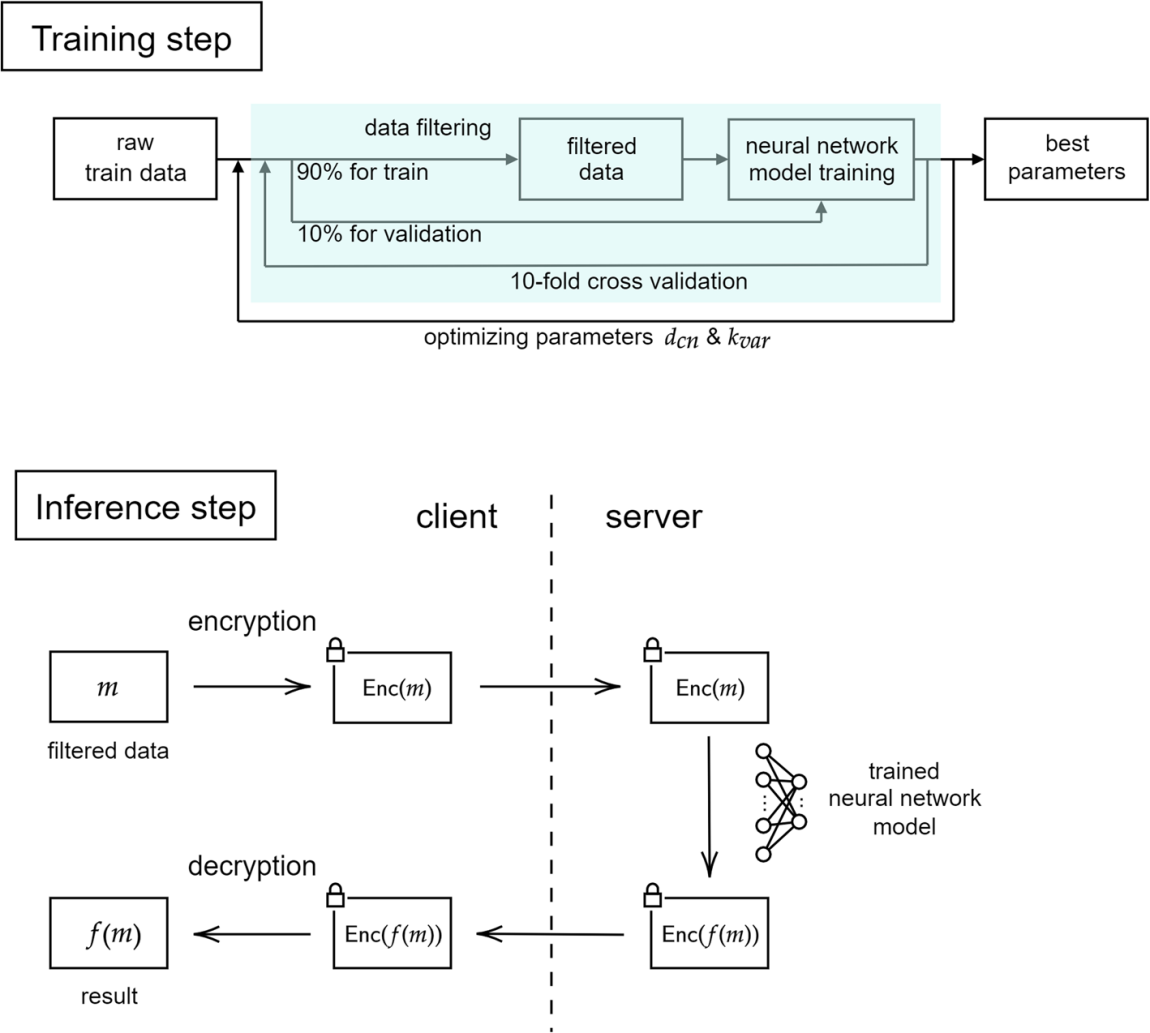
Illustration of our scenario for training and inference steps. In training step, our data filtering method reduces the size of raw data dramatically. Then, we use 10-fold cross validation to train shallow neural network (SNN) model using filtered data. As a result, we get the optimized parameters for both filtering and SNN model. In inference step, the protocol works as in the scenario using HE introduced in Background section. Since we use filtering method in preprocessing, only some chosen genes are needed to be encrypted in ciphertext. Then, we run HE-friendly SNN algorithm to evaluate our model. As a result, the client can receive the evaluated value using their private data without revealing any information

The detailed explanation of our scenario and method is illustrated in Fig. 1. Our protocol can be performed by using HE while ensuring client’s data privacy. Let *m* be the client’s private data and *f* be a function that computed the whole process of the model generated by the server. First, the client encrypts *m* by her own secret key and sends the ciphertext *Enc*(*m*) to server. Then, server evaluates his model using HE operations, so that he obtains the encrypted evaluation output *Enc*(*f*(*m*)) and sends it back to the client. Finally, the client performs HE decryption for *Enc*(*f*(*m*)) with the secret key to get the desired output *f*(*m*). Since the server can only access encrypted data, our protocol can achieve the desired security even on malicious servers.

In our implementation, all ciphertexts have a 128-bit security^1^ level regardless of the remained number of operations. This means that the ciphertext will not leak any information in a lifetime even if all the computing power in the world is used. Our ciphertext is based on the Ring-Learning with Error (LWE) problem which is attracting attention as it seems secure although quantum computers are developed. And we set the HE parameters to achieve 128-bit security estimated by Albrecht’s LWE estimator[38].

### Goal of this work

Our goal is to perform privacy-protecting machine learning computations on encrypted tumor datasets. In general, there are two main obstacles to achieving this goal. The first point is the size of dataset. Most HE schemes that support multi-encoding are able to contain at most 2^16^ or less number of data in a ciphertext with a practical parameter. Since typical genomic dataset consists of large-scale matrix with millions of elements, a large number of ciphertexts are required to encrypt data and a huge computational overhead occurs in the operation. Therefore, a preprocessing process that reduces the size while maintaining the properties of the initial data is essential, and because of the security problem, this method should be performed in a HE-friendly manner.

The second point is that the machine learning model should be performed using HE. Since the operations allowed in HE is limited, especially addition and multiplication, most of deep and complex models cannot be implemented using HE in practical time now. Even in simple cases such as single-layer neural networks, since most activation functions use non-arithmetic operations such as comparison (ReLU) or exponential functions (sigmoid, softmax), additional time consuming and accuracy loss occurs in the process of computing approximation of these functions with polynomials.

## Results

### Summary of results

In this paper, we propose a privacy-preserving multi-label classifier using a shallow neural network with a softmax activation function based on HE, which is also an outstanding solution of the first track of the iDASH 2020 competition. Our method is a supervised parallel integration method that uses unsupervised feature selection (cluster analysis) which provides the scalability on the parameter depending on the computational bound of HE. Our ideas can be categorized into three main subjects. First, we suggest data filtering method to reduce the size of the large raw data. This method is suitable for our scenario using HE because the data owner does not require additional operations other than filtering before encryption. Second, we modify the data packing method for matrix-vector multiplication [15] by duplicating the data in encryption to make a trade-off between the number of ciphertexts for packing and the number of rotations to reduce computational cost for HE. In multiplication between encrypted matrix and plain vector, the number of rotations occupies the most time cost, so we minimize the total time by choosing an appropriate number of copies in the encryption step. Lastly, we use elementary exponential approximation method to evaluate the microAUC value. We minimize the loss of the approximated microAUC from the real value not by focusing on minimizing the error in the approximation of the exponential function, but by using an approximation that shares properties with the exponential function.

### Dataset description

Our scenario focuses on the dataset originated from The Cancer Genome Atlas (TCGA) database, which is widely used in genomic research. The data of the somatic Single Nucleotide Variation (SNV) and gene-level Copy Number Variation (CNV) information for TCGA samples are downloaded from publicly available datasets [39] and [40], respectively. The training and testing data are generated based on the sample metadata downloaded from the TCGA project (available on 8/13/2020) and extracted the cases for all available cancer types. The samples first are filtered for the ones that exist in both SNV and CNV datasets. For each of the remaining cancer types, the data were randomly divided into training and testing datasets with 75% and 25% of all samples for the corresponding cancer type, respectively. Cancer types with less than 100 training samples are filtered out, and the resulting dataset consists of cancers from 11 sites: Bladder, Breast, Bronchus and Lung, Cervix uteri, Colon, Corpus uteri, Kidney, Liver and Intrahepatic bile ducts, Ovary, Skin, and Stomach.

The dataset consists of total 3,622 samples (2,713 for train, 909 for test) and 25,128 genes, and each sample has one cancer type out of 11 types of cancer, consists of two types of data: Copy Number (CN) data and Variants data. The dataset is available in [41] which is generated in the same way with iDASH 2020 competition track I and the details of dataset including the number of samples and the number of mutation’s effects for each feature are disclosed in Table 2.

**Table 2:** Sample and mutation statistics of the dataset on 11 cancer types. Note that the total number of genes are 25,128 and the number in Mutation’s Effect column means that the number of non-zero values in Variants dataset (which is less than *#* of samples ×*#* of genes)

Cancer Site	Samples (Train/Test)	Mutation’s Effect (Train/Test)			
		LOW	MODERATE	MODIFIER	HIGH
Bladder	258 / 87	25,641 / 7,261	61,862 / 17,386	11,303 / 3,181	9,616 / 2,500
Breast	201 / 67	11,915 / 4,591	30,966 / 13,107	8,335 / 3,450	7,828 / 6,149
Bronchus / Lung	638 / 213	61,277 / 21,114	166,945 / 57,898	28,039 / 9,590	25,819 / 9,089
Cervix uteri	149 / 50	12,084 / 4,608	28,515 / 10,913	14,715 / 5,284	4,278 / 1,751
Colon	256 / 86	42,501 / 11,410	105,179 / 26,225	29,525 / 7,484	24,320 / 6,653
Corpus uteri	219 / 73	139,405 / 32,877	364,241 / 87,297	167,526 / 41,800	60,846 / 16,427
Kidney	149 / 50	5,425 / 1,794	13,772 / 4,697	3,684 / 1,185	3,155 / 1,025
Liver / Intrahepatic bile ducts	189 / 64	7,198 / 2,687	19,697 / 6,946	6,186 / 2,219	3,149 / 1,132
Ovary	151 / 51	6,477 / 2,808	17,218 / 6,811	3,569 / 1,311	3,663 / 1,020
Skin	254 / 85	107,923 / 40,326	197,015 / 72,248	34,699 / 13,123	22,051 / 7,830
Stomach	249 / 83	32,972 / 13,131	78,593 / 30,874	14,630 / 5,910	20,571 / 8,087
Total	2,713 / 909	452,818 / 142,607	1,084,003 / 334,402	322,211 / 94,537	185,296 / 61,663

CN data consists of the copy number of the genes. The copy number data show the copy number variations (CNVs) of the genes as numbers, representing the state of the gene on the corresponding sample, whether the gene has duplication or deletion of a considerable number of base pairs. The data consist of 5 different levels 0, ±1,±2 where the negative and positive values represent deletion and duplication of the corresponding gene from the sample, respectively. Copy number with 0 means that the gene has no considerable variation on the corresponding gene from the sample.

Variants data consists of mutation data of selected pairs of samples and genes for each tumor type with various features: gene’s location on chromosomes, mutation type, whether the mutation is Single Nucleotide Polymorphism (SNP), and mutation’s effects separately predicted by two different methods.

### Neural network model and parameter selection

For the training step, we first downsize each sample by using our proposed filtering algorithms, and used it for the training of our neural network model. More precisely, we feed the downsized samples into our shallow neural network model, which consists of one hidden layer with 64 nodes and linear activation function and output layer with 11 nodes. During the training step, we used batch size of 32, number of epochs of 50 and dropout rate of 0.9.

To suitably use the trained model in downstream tasks over encrypted data, we varied (*d*_*cn*_,*k*_*var*_), the parameters for CN and variants dataset respectively, until the size of downsized data are less or equal to 2^*B*^ for each *B* from 9 to 12. In our algorithm, the size of model is determined by the size of filtered data under two parameters. For each *B*, we seek the best pair of parameters, *d*_*cn*_ among {0,0.01,⋯,0.24} and *k*_*var*_ among {0,10,⋯,590}; more precisely, we use 10-fold cross validation to find the (*d*_*cn*_,*k*_*var*_) pair with best microAUC such that the size of model is less or equal to 2^*B*^. After selecting the parameters, *d*_*cn*_ and *k*_*var*_, we train the model on the entire training dataset, and feed it into the inference step over encrypted data.

For the inference step over encrypted data, we adopt HEaaN library, the implementation of CKKS scheme [14]. The CKKS parameters are chosen by the ring dimension 2^17^ and the ciphertext modulus 2^2670^. We used signed binary secret, which satisfy more than 128-bit security according to Albrecht’s LWE estimator [38]. For the scal- ing factor of CKKS, we choose the scaling factor by 2^60^ for ciphertexts and 2^40^ to encode plain vectors for constant multiplication or masking vectors.

For the approximation of Softmax, we use Goldschmidt algorithm with *M*=80 and *d*=30 for the inversion, and for the exponential function, we use (*r*,*L*)=(4,32) for *B*=9,10 and (4,64) for *B*=11,12, respectively. All experiments were performed on Intel Xeon CPU E5-2620v4 at 2.10GHz processor and used 8 threads. The detailed description of CKKS parameters is stated in Methods section and we refer Algorithm 4 for the parameters used in our algorithms.

Our codes for the training step can be found in https://github.com/jaihyunp/iDash2020, and those for the inference step can be found in docker repository swanhong/idash2020.

### Experimental results

#### Training model with plain dataset

**Fig. 2:**
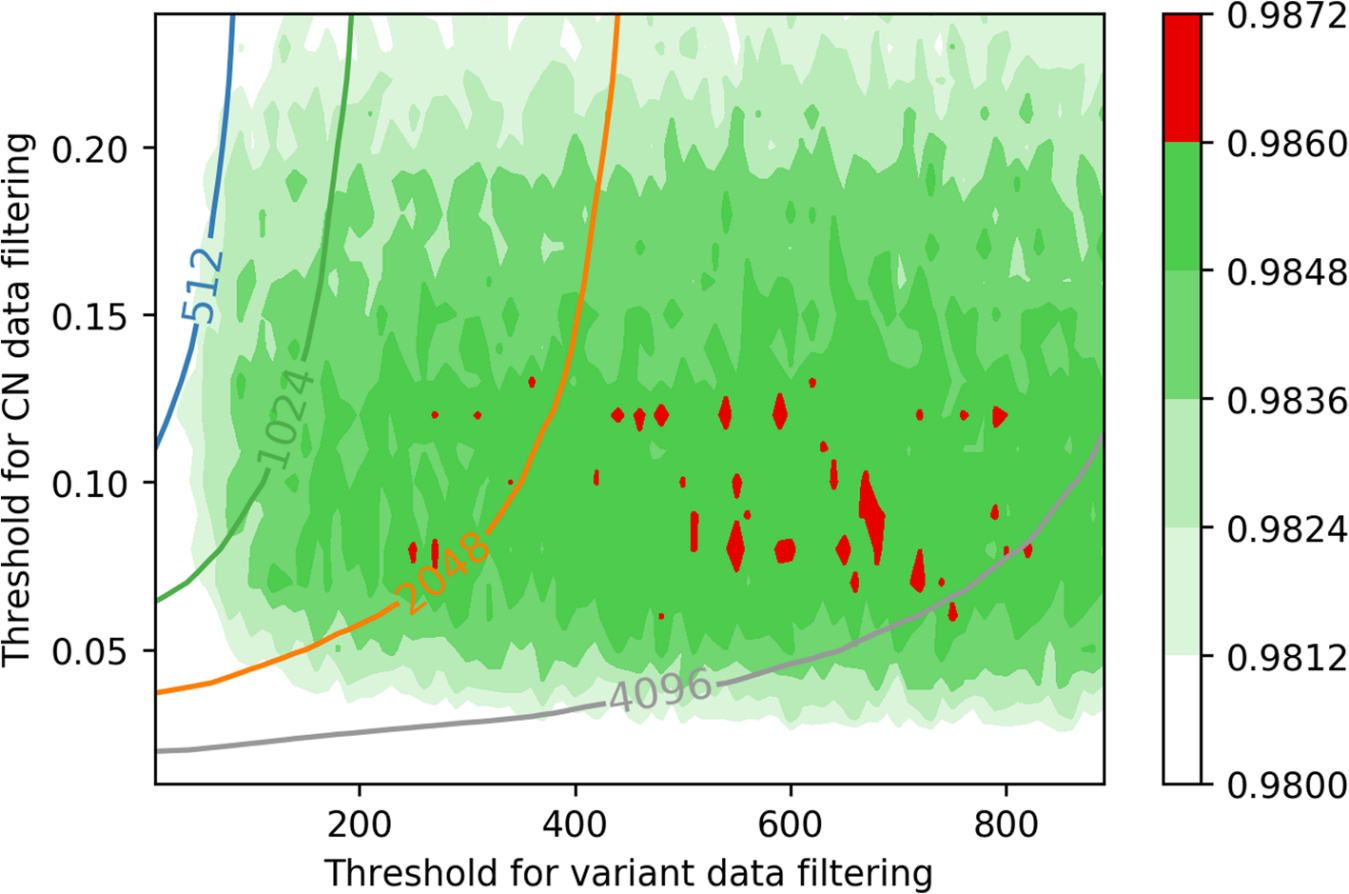
Illustration of microAUC of each model during 10-fold cross validation with given pair of (*d*_*cn*_,*k*_*var*_). The model tends to show a good performance on *d*_*cn*_≈0.08, and larger *k*_*var*_ shows a better microAUC near *d*_*cn*_=0.08. As we visualize in the graph with contours, larger *d*_*cn*_ and *k*_*var*_ accompanies a larger size of the model

**Fig. 3:**
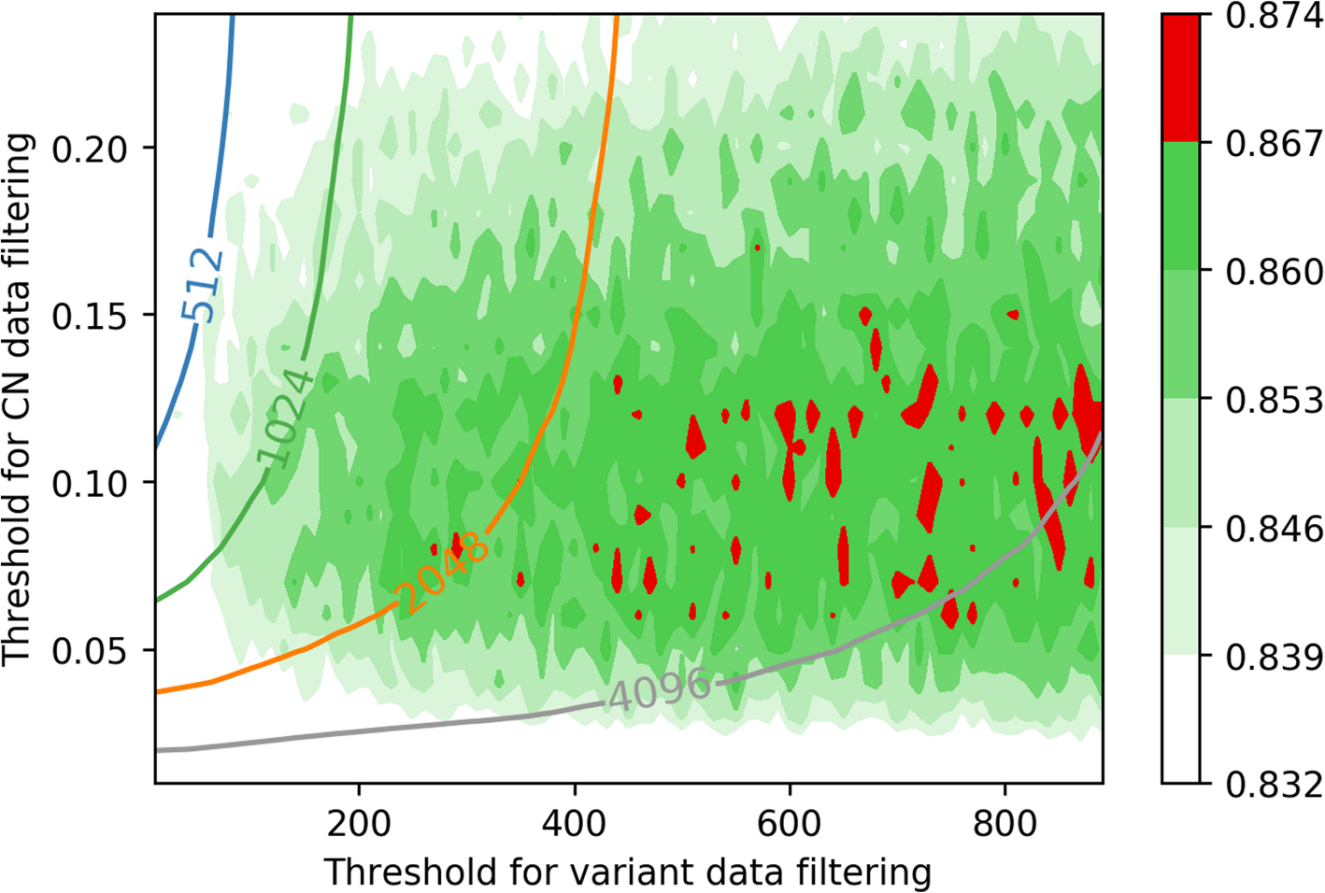
Illustration of accuracy of each model during 10-fold cross validation with given pair of (*d*_*cn*_,*k*_*var*_). The model tends to show a good performance on 0.05≤*d*_*cn*_≤0.15, and larger *k*_*var*_ shows a better microAUC. As we visualize in the graph with contours, larger *d*_*cn*_ and *k*_*var*_ accompanies a larger size of the model

**Fig. 4:**
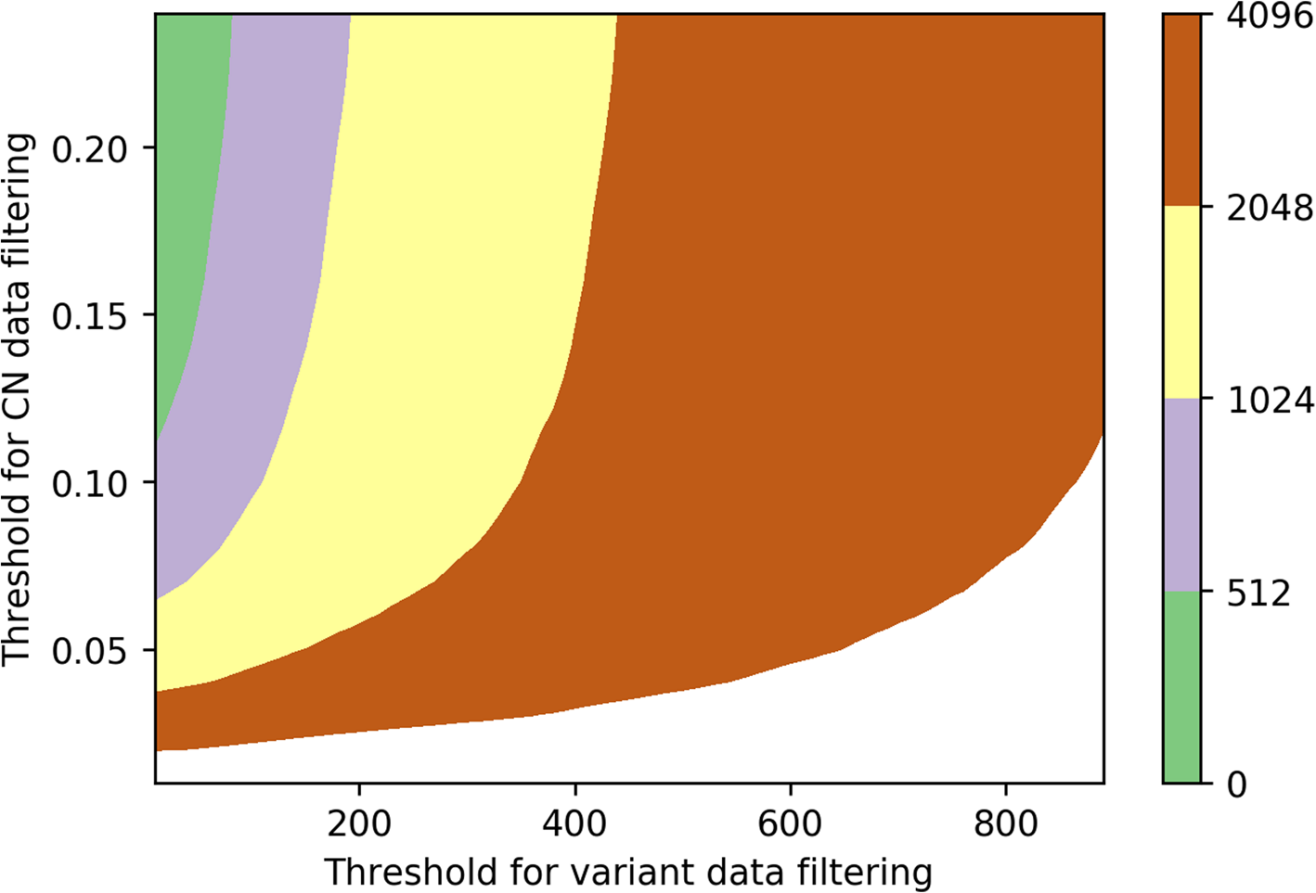
Illustration of the size of each model trained on the entire training set with given pair of (*d*_*cn*_,*k*_*var*_). To optimize the computational cost on the privacy-preserving inference step based on homomorphic encryption, we seek the best parameters (*d*_*cn*_ and *k*_*var*_) with the model sizes of less or equal to 2^*B*^ for each *B*=9,10,11, and 12

Gradually changing two parameters, our results are presented in Figs. 2 and 3. Figure 4 visualizes how (*d*_*cn*_,*k*_*var*_) pair determines the size of model. The model tends to show the best performance in terms of microAUC on *d*_*cn*_≈0.08, and larger *k*_*var*_ tends to show a better performance. However, to optimize the computational cost on the privacy-preserving inference step based on homomorphic encryption, we seek the best parameters (*d*_*cn*_ and *k*_*var*_) for the model of size less or equal to 2^*B*^ for each *B*=9,10,11, and 12.

The best (*d*_*cn*_,*k*_*var*_) pair for each size of model, 2^*B*^, and its performance on 10-fold cross validation is given in Table 3.

**Table 3:** The performance of correctness with the best pair of (*d*_*cn*_,*k*_*var*_) for each threshold of the size of the model among training dataset. The best pairs are chosen from the result in Fig. 2 that shows best microAUC score in the same thresholds. We note that 10-fold cross validation is used on the training set

Threshold of the model size	*d*_*cn*_	*k*_*var*_	Filtered genes		microAUC	Accuracy
			CN	Variants		
512	0.17	60	243	265	0.98179	0.83321
1024	0.13	90	358	404	0.98523	0.84317
2048	0.08	270	709	1198	0.98625	0.86827
4096	0.08	550	709	2364	0.98704	0.86974

#### Encrypted inference results

**Fig. 5:**
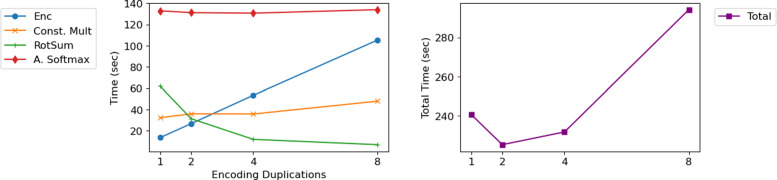
Illustration of time cost for each duplication numbers when the threshold of the model size is 1024 (*B*=10). As encoding duplication number increases, the time cost for Encryption increases linearly while the cost for *RotSum* decreases approximately by log scale, and the other parts are stationary. In our implementation environment, the total time cost is minimal when duplication number is 2

Using filtered gene in preprocessing step and the model built in training step, we compute inference step over encrypted data as stated in previous sections. As a result, we estimate the time cost for each round-trip step including encryption, constant multiplication, rotate and summation, Approximate softmax evaluation, and decryption step. The results are stated in Table 4. In the table, the column *Encoding Duplication* means the parameter *m* that we used as the number of duplication in encryption. As *m* increases, the number of required ciphertexts increases linearly so the time cost for encryption also similarly increases and the number of rotations decreases as $O\left (\frac {1}{m}\log \frac {1}{m}\right)$ scale. With such trade-offs, denoting the bound of the number of genes by 2^*B*^, we notice that the time cost is minimized when *m*=4 for *B*=9,*m*=2 for *B*=10,11, and *m*=4 for *B*=12, respectively. We illustrate the scalability of time cost from *m* in Fig. 5 for *B*=10. Moreover, we state the final microAUc value and accuracy for each *B* in the table. In summary, we get about 0.988 microAUC and 85% accuracy for test dataset, which is the relatively better result compared to other works using the dataset from same data source [4, 26].

**Table 4:** Table of experiment results for inference step over encrypted state. For each threshold for the model size, the number of chosen genes for CN and variants data are as in Table 3. Encoding Duplication means the number of duplications for *X* in encoding step, refers the parameter *m*. For each case, we state the microAUC value and accuracy on the right columns. These results come from the our trained model with test dataset

Threshold of the model size	Encoding Duplication	Computation Time (sec) Accuracy							
		Encryption	Const. Mult	RotSum	A. Softmax	Decryption	Total		
512	1	6.7	16.8	61.0	130.5	0.1	215.1	0.9866	84.60
	2	13.4	17.8	29.6	131.0	0.1	191.9		
	4	26.5	17.5	11.9	132.9	0.1	188.9		
	8	52.6	23.8	6.5	133.0	0.1	216.0		
1024	1	13.5	32.3	62.0	132.9	0.1	240.8	0.9882	85.15
	2	26.7	36.0	31.4	131.2	0.1	225.4		
	4	53.4	35.8	11.9	130.7	0.1	231.9		
	8	105.4	47.9	6.9	134.0	0.1	294.3		
2048	1	26.7	64.9	63.2	132.1	0.1	287.0	0.9857	86.47
	2	54.0	70.5	29.3	131.4	0.1	285.3		
	4	106.8	72.2	13.2	131.6	0.1	323.9		
	8	211.5	98.1	8.3	133.9	0.1	451.9		
4096	1	53.8	129.4	63.6	130.7	0.1	377.8	0.9862	86.25
	2	107.7	143.1	31.3	135.1	0.1	417.6		
	4	213.9	152.6	15.2	140.5	0.1	522.3		

## Discussion

Our work in this paper was awarded fist place (with other teams including Desilo, Inpher and SamsungSDS) in the HE track of iDASH 2020 competition. Unlike other award winners who obtained relatively low microAUC values (close to 0.95) by performing linear activation within a very short time, our result shows a high microAUC value by proposing the only method for applying softmax activation within a practical time. In addition, our preprocessing method has scalability, making it possible to obtain a higher microAUC value through additional time consumption, which is different from other teams.

The main purpose of our data preprocessing is to remove irrelevant genes in the data. The genes removed have a similar effect to the remaining genes, or are have a weak effect on the cancer type classification. CN data filtering extracts the genes with the hamming distance of the samples’ copy number, based on the fact that the adjacent genes have copy number similarity. This filtering technique can be applied to other types of the genetic dataset with an appropriate threshold *d*_*cn*_ for each dataset. Variants data filtering can be also applied to other types of datasets that have a lot of empty information. Since various genetic datasets are ‘empty-sparse’, not sparse with lots of 0s but no information, our filtering technique can be used to efficiently extract the relevant features. However, since our method focused only on the filtering method to select meaningful genes, it cna be expected that the high microAUC values can be obtained through other methodologies that perform simple operations. In future studies, combining our method with other HE-friendly preprocessing methods [4, 26] would be able to show better accuracy using the same dataset.

To our best knowledge, our softmax approximation algorithm is the first approach to use the softmax activation function for the neural network implemented with HE. However, the size of the input data must be adjusted through normalization, and it should be preceded that the process of predicting the range of the maximum and minimum values of the matrix product *XW* as a result of the train data to compute the approximation of exponential function and the Goldschmidt algorithm. In addition, our algorithm may not be suitable for computing deep neural network models with multiple softmax activation functions for a limited time, as it consumes a lot of HE depth in approximating the softmax. In further research, we expect that other popular activation functions, such as sigmoid or ReLU, can be combined with our neural network model to improve the final score.

## Conclusions

In this paper, we propose the first result of privacy-preserving multi-label classification for tumor data using neural network with softmax activation. To enable implementation using HE within a practical time, we suggest filtering method to reduce the size of genes from 25,128 to pre-fixed bound 2^*B*^ where *B* is in {9,10,11,12}. For encoding filtered dataset to ciphertexts, we suggest the duplicating method that encodes same data multiple time, which decreases the time cost for matrix-vector multiplication for evaluating neural network model and provides a time cost trade-off between encryption step and message rotation step. Also, our approximation method for the softmax function enables to apply the softmax activation function for shallow neural network model. As a result, we obtain inference results with microAUC values of about 0.988 to classify multi-label tumor data in 5 minutes. If the time given for the inference step is not limited, our preprocessing and approximation methods can be used as building blocks for general deep neural networks.

## Methods

### Notations

All logarithms are base 2 unless otherwise indicated. The vectors are denoted with upper arrow. We denote an entry of the vector by using the same character with index. We denote Hadamard multipcation between two vectors by $\vec {a} \odot \vec {b}$. Also, for simplicity, we denote the [*x*]_*n*_ be the number in {1,⋯,*n*} satisfying [*x*]_*n*_=*x* mod *n*.

For a matrix *A*, *A*(*i*,*j*) means the *i* th row and *j* th column element of *A*. Also, we denote the submatrix of *A* with *i*∈*I* th rows and *j*∈*J* th columns from *A* by *A*[*I*,*J*]. In this case, the colon(:) indicates the whole index set.

### Approximate homomorphic encryption

Since HE enables anaylsis of encrypted data while preserving the privacy of message from operators, it is considered as one of the beneficial tool to delegate operations that requires sensitive data without revealing any information. Unlike other popular cryptographic tools including multi-party computation, which require protocol participants to continuously interact to each other through the scenario, HE has the advantage that no additional actions or online processes are required for the message owners after they encrypts and transmits data to the operator. Through these characteristics, HE has been exploited in various field such as ML that requires computation using sensitive information such as genomic data [35, 42, 43] or financial data [44]. Additionally, HE can also play an important role in the protection of data in the computation process in applications that require computations between real data, such as ML [45] or cyber physics system [46].

Since Gentry firstly suggested in his blueprint [47] in 2009, a number of HE schemes have been proposed to achieve useful properties for applications. Each scheme has advantages in operations in a particular message space, such as finite field operations [12, 36] or boolean circuit [37]. However, many well-known deep learning algorithms [48, 49] cannot be directly implemented because of some limitations of HE. First, since most HE methods only support multiplication operations less than fixed number of depths, it is difficult to implement a method using a large number of layers in a neural network. Therefore, most HE applications focus on implementing shallow neural networks or logistic regression. Although it is possible to recover the depth of the ciphertext through an operation called bootstrapping, it requires a very high computational overhead compared to other basic operations. Second, each scheme does not support both common arithmetic operations and binary(or logistic) operations at the same time. While many ML algorithms require both operations such as matrix-vector multiplication or ReLU function, the application of the HE scheme requires the way to efficiently perform unfavorable operations.

The approximate HE scheme, namely as CKKS scheme, is proposed by Cheon et al. [14]. The main feature of approximate HE scheme is that it deals with operations in complex numbers $\mathbb {C}$ and it supports fixed-point arithmetic operations between encrypted data. It also supports approximate arithmetic, which considers the noise of the ciphertext as part of the message to increase the efficiency of the operation. Since most ML algorithms mainly use fixed-point operations on real data or noise-friendly algorithms such as gradient descent, CKKS scheme takes advantages of the most of ML applications compared to other HE schemes.

We remark that the word *approximate* does not mean that the homomorphic operations contain large errors and result in data loss, but rather that the very small errors are allowed in the message to increases efficiency of the operations in the scheme. In machine learning applications, such errors does not ruin the message as the scheme guarantees a sufficiently large precision if practical parameters are used.

For the rest of this section, we formally describe CKKS scheme. Let *L* be a level parameter that a fresh ciphertext is equipped with a modulus *q*_*L*_=(2^*Δ*^)^*L*^, and *q*_*ℓ*_:=(2^*Δ*^)^*ℓ*^ for 1≤*ℓ*≤*L* for some scaling factor *Δ*. Let $R:=\mathbb {Z}[X]/(X^{N}+1)$ be a cyclotomic ring for a power-of-two *N* and *R*_*q*_ be a modulo-*q* quotient ring of *R*, i.e., *R*_*q*_=*R*/*qR*. The distribution *χ*_*enc*_ and *χ*_*err*_ denote the discrete Gaussian distribution with some fixed standard deviation. The distribution *χ*_*key*_ outputs a polynomial of {−1,0,1}-coefficient. We denote the rounding function ⌊·⌉ and modulo-*q* operation [·]_*q*_.

CKKS scheme uses a plaintext vector $\vec {m}\in \mathbb {C}^{N/2}$ and provides enrty-wise operation, called Single-Instrument-Multiple-Data (SIMD) operation, such as addition, substitution, and Hadamard multiplication between vectors. To encrypt complex value, CKKS uses a field isomorphism $\tau : \mathbb {R}[X]/(X^{N}+1)\rightarrow \mathbb {C}^{N/2}$ called canonical embedding.

- KeyGen(*params*).
  - Sample *s*←*χ*_*key*_ and Set the secret key as *sk*=(1,*s*).
  - Sample $a\leftarrow U(R_{q_{L}})$ and *e*←*χ*_*err*_. Set the public key as $\mathsf {pk} = (b,a)\in R_{q_{L}}^{2}$ where $b=[-a\cdot s + e]_{q_{L}}$.
- Enc_pk_(m). Given a message m∈*R*, sample *v*←*χ*_*enc*_ and *e*_0_,*e*_1_←*χ*_*err*_. Output the ciphertext $\phantom {\dot {i}\!}\mathsf {ct} =[v\cdot \mathsf {pk} + (\mathsf {m}+e_{0},e_{1})]_{q_{L}}$.
- Dec_sk_(ct). Given a ciphertext $\mathsf {ct}\in R_{q_{\ell }}^{2}$, output m^′^=〈ct,sk〉.
- Add/Sub(ct,ct^′^). Given two ciphertext $\mathsf {ct}, \mathsf {ct}' \in R_{q_{\ell }}^{2}$, output the ciphertext $\phantom {\dot {i}\!}\mathsf {ct}_{add}/\mathsf {ct}_{sub} = [\mathsf {ct} \pm \mathsf {ct}']_{q_{\ell }}$ encrypting a plaintext vector $\vec {m}_{1} \pm \vec {m}_{2}$.
- Mult_evk_(ct,ct ^′^). Given two ciphertexts $\mathsf {ct}, \mathsf {ct}' \in R_{q_{\ell }}^{2}$, output a level-downed ciphertext $\mathsf {ct}_{mult}\in R_{q_{\ell -1}}^{2}$ encrypting a plaintext vector $\vec {m}_{1}\odot \vec {m}_{2}$.
- Rot_*rk*_(*ct*;*r*). For a ciphertext *ct* encrypting a plaintext vector $\vec {m} = (m_{1},\cdots,m_{n})$, output a ciphertext *ct*^′^ encrypting a plaintext vector $\vec {m}' = (m_{r+1},\cdots,m_{n},m_{1},\cdots,m_{r})$ which is the (left) rotated plaintext vector of *ct* by *r* positions.

For the remind of the paper, we may denote the operations between ciphertexts or ciphertext and plain vector by common symbols, such as *Add*(*ct*_1_,*ct*_2_)=*ct*_1_+*ct*_2_ or $\mathsf {CMult}(\mathsf {ct}, \vec {c}) = \mathsf {ct} \cdot \vec {c}$ for simplicity.

### Data preprocessing

**Fig. 6:**
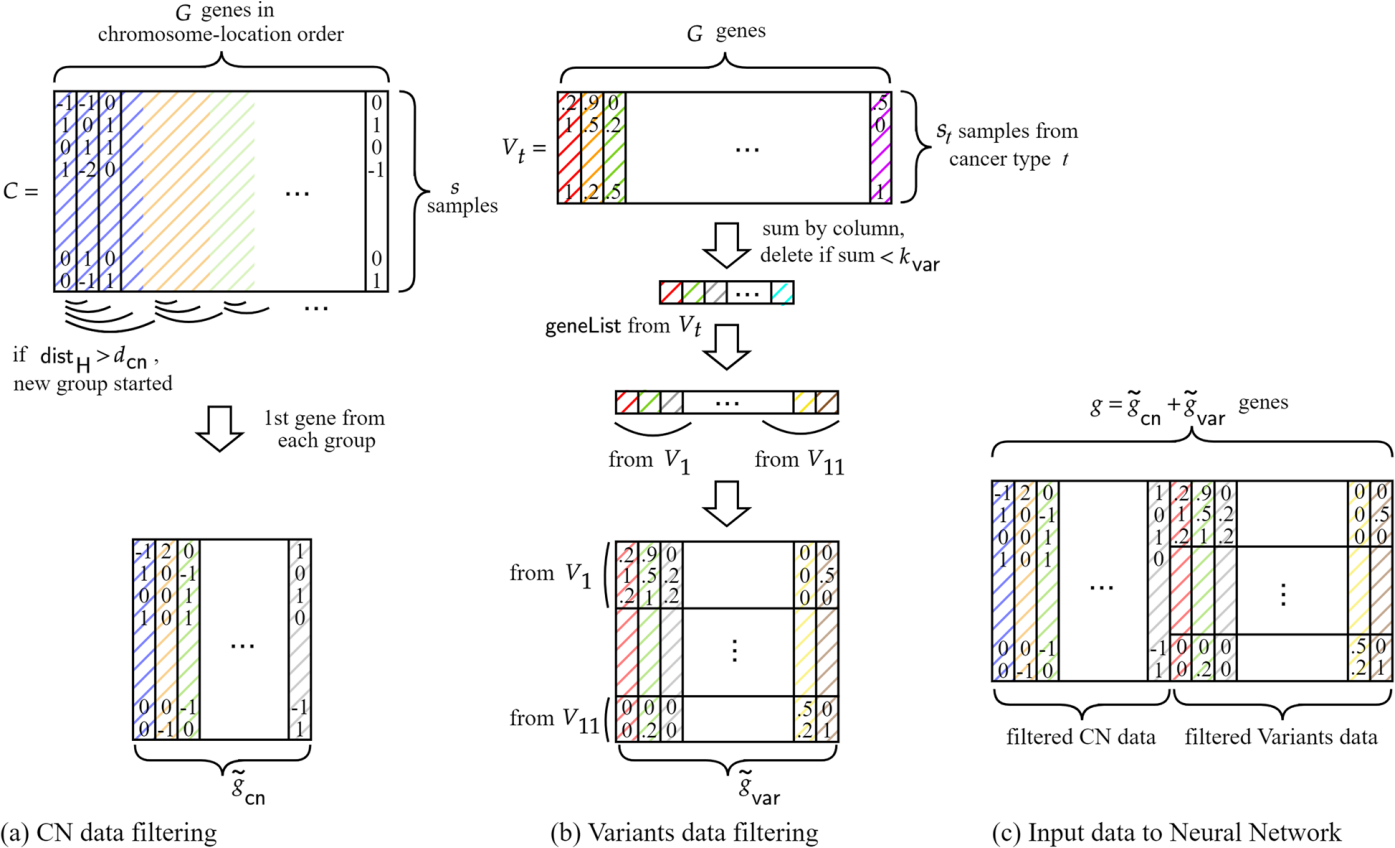
Workflow of the data preprocessing. (a) CN data filtering. CN data matrix *C* consists of *s*=2713 samples and *G*=25128 genes, where each entry represents the copy number of the corresponding sample and gene in 5 levels: 0, ±1,±2. The CN data filtering is based on the copy number similarity of adjacent genes. (b) Variants data filtering. Variants data matrix *V*_*t*_ for each tumor type *t*=1,2,⋯,11 consists of mutations’ effect data. The data is encoded to real values 0,0.2,0.5,0.9, and 1.0 where 0 is for the entry with no information. The Variants data filtering removes irrelevant genes with ineffective mutations. (c) Input data to Shallow Neural Network. The filtered CN and Variants data is concatenated and input to our Neural Network

Given more than 25,000 genes, meaningful gene selection is essential for efficient ML using HE. Therefore, we introduce a new data preprocessing technique consisting of a feature selection method modified from [4] to obtain significant genes useful for tumor prediction. Our data preprocessing method consists of two filtering algorithms that extract specific features from two different data types. The resulting filtered data matrix is concatenated as shown in Fig. 6(c) and inserted into our neural network.

In the rest of paper, we denote the number of samples and genes from original data by *s* and *G* and the number of types of tumor by *T*. In our dataset, these notations contain values *s*=2,713(train) or 909(test), *G*=25,128 and *T*=11. Using the abstract notation, the CN and Variants data can be understood as a matrix of the size *s*×*G* and the *T* number of set of information. Since the number of genes *G* is too large to handle with HE efficiently, we propose the preprocessing method which is easy to compute both client-side and server-side in our scenario to reduce the number of filtered gene less than fixed bound.

#### CN data filtering

Our main approach for CN data filtering is to filter out the irrelevant genes with cluster analysis based on the similarity of the neighboring genes. Modified from CGF in [4], we introduce a new cluster analysis using hamming distance instead of Jaccard distance. To reduce the number of input features we take only one gene from each cluster, unlike CGF.

Let *C*∈{0,±1,±2}^*s*×*G*^ be the matrix of copy number data, where the *s* rows correspond to the *s* samples, and the *g* columns correspond to *G* genes; *C*(*i*,*j*) is a copy number that corresponds to gene *j* of sample *i* (see Fig. 6(a)). We initially sort the raw CN data according to the order of the gene positions on the chromosome.

Then, we cluster the genes into groups by their copy number similarity (line 3-10 in Algorithm 1). For two gene columns $\vec {p}, \vec {q} \in \{0, \pm 1, \pm 2\}^{1 \times G}$, we use the Hamming distance as the similarity measure

1 $$\begin{array}{@{}rcl@{}} \mathsf{dist}_{\mathsf{H}}(\vec{p}, \vec{q}) = \text{the number of }i\text{ such that}\ p_{i} \neq q_{i} \end{array} $$

where *p*_*i*_ and *q*_*i*_ are the *i* th entry of vectors $\vec {p}$ and $\vec {q}$, which represents the copy numbers of each gene for *i*th sample. Starting from the first gene in *C*, say a representative of the first group, we calculate the hamming distance with the following genes in order. Until the distance is less than the predetermined threshold *d*_*cn*_, we merge each corresponding gene to the first group. If the distance first reached the threshold *d*_*cn*_, the first group ends and the corresponding gene becomes a representative of the second group. Then start from the new representative, calculate the hamming distance with the following genes.

Repeating this algorithm until the genes are all clustered, each gene is in a unique group with neighboring genes. Finally, we filter the CN data with the representative genes, the first gene in each group, resulting in a matrix $\tilde {C} \in \{0, \pm 1, \pm 2\}^{s \times \tilde {g}_{\mathsf {cn}}}$ where $\tilde {g}_{\mathsf {cn}}$ is the number of groups as the result of our filtering. Note that $\tilde {g}_{\mathsf {cn}}$ is much smaller than the number of original genes. We formally state our CN data filtering algorithm in Algorithm 1 and shown in Fig. 6(a).


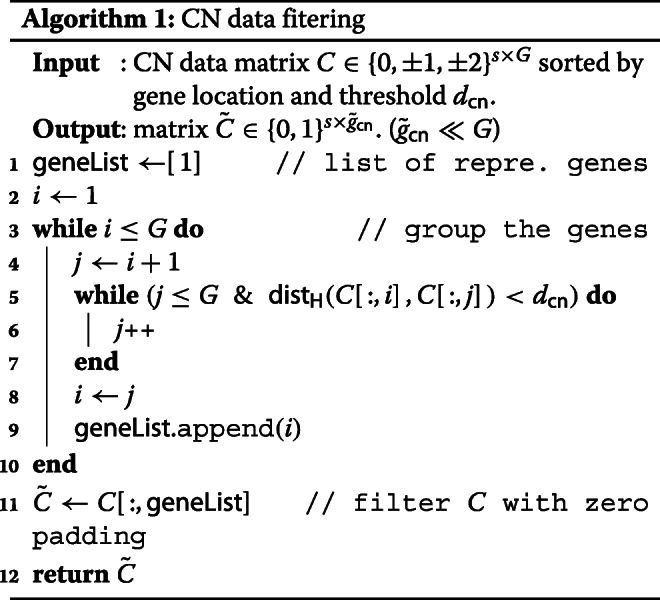


#### Variants data filtering

For Variants data filtering, the feature selection mechanism uses only a mutation’s effect data classified in 4 different levels: LOW, MODERATE, MODIFIER, and HIGH. The Variants data filtering works based on the effectiveness of the mutations. It filters out the genes with less effective mutations.

In the Variants data, for each sample, only a few mutation effect data of individually selected genes are given. So the raw Variants data for whole samples and genes is almost empty, which cannot be used immediately as a neural network input. Hence our Variants data filtering is separately applied to each cancer type to reduce the empty spaces and fill in yet remaining spaces with zero.

Let *V*_*t*_ be the matrix of the raw data with mutation’s effect corresponding to the tumor type *t* (1≤*t*≤*T*). *V*_*t*_ is a *s*_*t*_×*G* matrix with mutation’s effect as strings, where *s*_*t*_ rows correspond to *s*_*t*_ samples and *G* columns correspond to whole *G* genes for each *t*. *V*_*t*_(*i*,*j*) is the effect of the mutation in *j* th gene of *i* th sample with *t* (see Fig. 6(b)). We first encode the string data to predetermined real values between 0 and 1: LOW = 0.2, MODERATE = 0.5, MODIFIER = 0.9, HIGH = 1.0, and 0 if no information exists. The resulting encoded Variants matrix is sparse matrix in $\phantom {\dot {i}\!}E^{s_{t} \times G}$, where *E*={0,0.2,0.5,0.9,1.0} be a set of encoding values.

Secondly, we sum *V*_*t*_ by each gene column and if the sum is more than the predetermined threshold *k*_*var*_, then put the gene in the list of the selected genes (line 2-8 in Algorithm 2). The column-wise summation $\sum _{i=1}^{s_{t}}V_{t}(i,j)$ is a mutation’s effect of *j*th gene to the *s*_*t*_ samples, so the selected genes in list can be regarded as genes with considerable mutation effects. The union of the selected genes from each tumor type *t* is the set of filtered genes.

Finally, we filter the whole Variants data with the filtered genes for each *t*, resulting in a matrix $\tilde {V} \in E^{s \times \tilde {g}_{\mathsf {var}}}$ with much smaller genes to whole samples The workflow of the Variants data filtering technique is shown in Fig. 6(b).


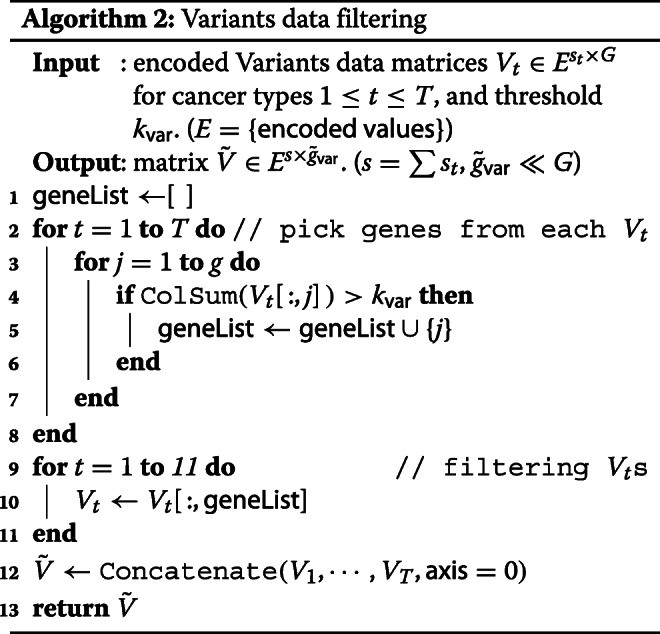


### Roadmap for training step over training data

After preprocessing for CN and variants data, we get the data matrix $X = [\tilde {C}|\tilde {V}]$ with size *s*×*g* where $g = \tilde {g}_{\mathsf {cn}} + \tilde {g}_{\mathsf {var}}$ by concatenating two filtered results. Then, with the tumor data *Y*, we train a neural network model from (*X*,*Y*) in plain while transform *Y* as the one-hot encoded label matrix with size *s*×*T*. Here, we note that *T* is 11 in our dataset, which is relatively small than *s* and *g*. Our neural network model consists of one hidden layer with 64 nodes and linear activation function and output layer with 11 nodes. In the output layer, we use softmax activation function to output their predicted value. During the training phase, we used Keras library [50] in Python with batch size of 32, number of epochs of 50 and dropout rate of 0.9. Note that we used sufficiently big dropout rate in order to avoid overfitting since the layers in our model are small.

To select the best parameters, *d*_*cn*_ and *k*_*var*_, for the preprocessing on the given train dataset, we train and evaluate shallow neural network models using 10-fold cross validation with filtered data based on each pair of parameters. After selecting the best parameters, we train the shallow neural network model on the entire train dataset using the best parameters, and we use the trained model for the inference step over encrypted test dataset.

### Roadmap for inference step over encrypted data

From now on, we state the method to compute inference step from our model with encrypted data. Precisely, input data matrix *X* with size *s*×*g* and weight matrix *W* with size *g*×*T* are given (recall that *s*,*g* and *T* refers the number of samples, genes, and tumors, respectively). Since *T* is relatively small than *s* and *g* in practice, we consider the matrix-matrix multiplication *Y*=*X*·*W* as matrix-vector multiplications $\vec {y}_{i} = X \cdot \vec {w}_{i}$ for 0≤*i*<*T*, where $\vec {w}_{i}$ is the (*i*+1)th column of *W*. Then, for each row *Y*_*j*_ of *Y* (0≤*j*<*s*), we compute the softmax function to get final score of our model. Since the matrix *Y* is still encrypted, we need to compute an approximate function of softmax. In short, our Method can be divided into three steps:

1. Data Packing : encrypt the matrix *X* to a number of ciphertexts {*ct*_*i*_}.
2. Matrix-Vector Multiplication : using {*ct*_*i*_} and *W*, compute matrix-vector multiplication to get the ciphertext *ct*_*Y*_ that contains *Y*=*X*·*W*.
3. Softmax Evaluation : compute approximate softmax function for *ct*_*Y*_ to obtain softmax output for each row of *Y*.

We break the method down into the first two steps and the other, and explain the main ideas in each section.

### Data packing and matrix multiplications

Although CKKS scheme supports SIMD operation between vectors, the SIMD operation cannot be directly applied to the matrix-vector multiplication operation. Therefore, in order to efficiently perform a matrix-vector multiplication operation using HE, a process of mapping a matrix to a number of vectors is required. In this section, focusing on the matrix multiplications in our scenario, we first state the naive approach and then suggests our optimization method.

**Fig. 7:**
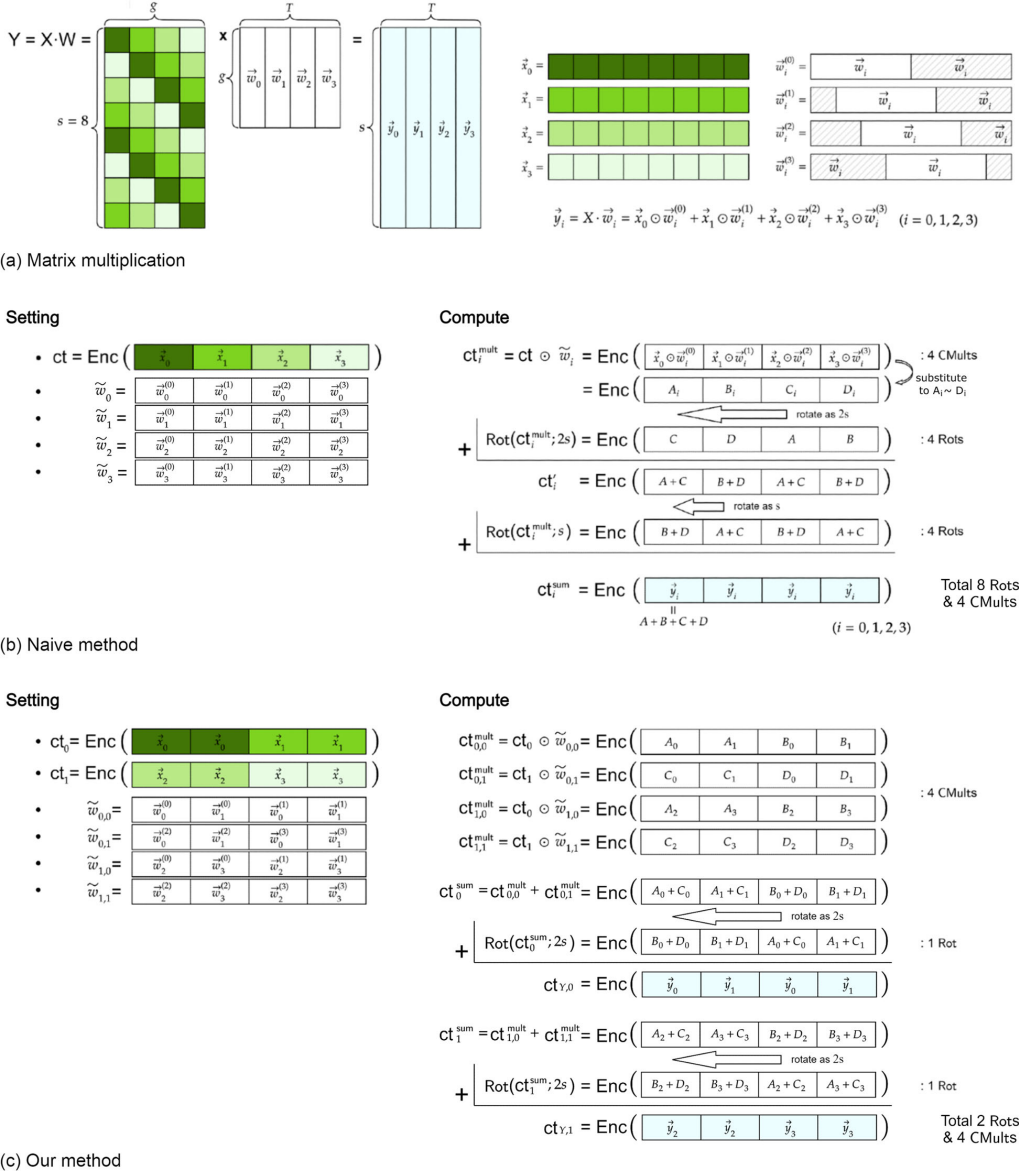
Illustration of data packing and matrix multiplication. To efficiently compute with encrypted data, data packing and matrix multiplication method should be highly concerned. (a) We basically follow the method in [15] which breaks the matrix-matrix multiplication into matrix-vector multiplications with vectors of special form. (b) Naïve packing method with 4 *CMult*s and 8 *Rot*s and (c) ours with 4 *CMult*s and 2 *Rot*s are given as a toy example with *s*=8 samples, *g*=4 genes, *T*=4 tumor types and ciphertext slot-size *n*=32, when using *m*=2 duplication of $\vec {x}_{i}$s (See *Warm-up with Toy Example*). If we use imaginary part for message space, we can even reduce the number of *CMult*s to $\frac {sg}{2n} \cdot m \lceil \frac {T}{m} \rceil = 2$, where the number of *Rot*s remains the same as $\log \frac {n}{ms} \cdot \lceil \frac {T}{m} \rceil =2$ (See *Putting It All Together*)

The main idea of mapping a matrix to vectors for this computation is suggested in [15], which is that $X \cdot \vec {w}_{i}$ can be understood as *n* times of slot-wise vector multiplication between *diagonal* part of *X* and *w*_*i*_. Concretely, we define $\vec {x}_{j}$ by the *j*-th diagonal part of *X* so that $\vec {x}_{j} = (X(k, [j+k-1]_{g})_{1 \le k \le s}$ for 0≤*j*<*g*. For the repeated rotation of each column $\vec {w}_{i}$ of *W*, we define with superscript with parentheses as $\vec {w}_{i}^{(j)} = (\vec {w}_{i}[j+k-1]_{g})_{1\le k \le s}$ for 0≤*j*<*g*. Then, the matrix-vector multiplication can be computed as $\vec {y}_{i} = X \cdot \vec {w}_{i} = \sum _{j=0}^{g-1} \vec {x}_{j} \odot \vec {w}_{i}^{(j)}.$ Hence, the multiplication can be done if we encrypt $\vec {x}_{j}$’s in several ciphertexts and compute constant multiplication between ciphertext and plain vector $\vec {w}_{i}^{(j)}$ (see Fig. 7(a)).

#### Warm-up with toy example

Before we state our explicit method for encodings and matrix-multiplications, we start with a toy example, simplifying the parameters as *s*=8,*g*=4, and *T*=4, and assumsing that the number of slots in one ciphertext is 4 times larger than the size of vector *s*. Then our goal is to compute $\vec {y}_{i} = \sum _{j=0}^{3} \vec {x}_{j} \odot \vec {w}_{i}^{(j)}$ for 0≤*i*<4. In the naive approach, we encode and encrypt $\vec {x}_{i}$’s by $\mathsf {ct} = \mathsf {Enc}(\vec {x}_{1} \| \vec {x}_{2} \| \vec {x}_{3} \| \vec {x}_{4})$ and define $\tilde {w}_{i} = (\vec {w}_{i}^{(0)} \| \vec {w}_{i}^{(1)} \| \vec {w}_{i}^{(2)} \| \vec {w}_{i}^{(3)})$ for each *i*. Then we can compute Hadamard multiplication by $\mathsf {ct}_{i}^{\mathsf {mult}} = \mathsf {ct} \cdot \tilde {w}_{i}$, which contains the message vector $(\vec {x}_{0} \odot \vec {w}_{i}^{(0)})\| \cdots \| (\vec {x}_{3} \odot \vec {w}_{i}^{(3)})$. Hence, the final sum is obtained by computing rotation twice as $\mathsf {ct}_{i}' = \mathsf {ct}_{i}^{\mathsf {mult}} + \mathsf {Rot}(\mathsf {ct}_{i}^{\mathsf {mult}};2s)$ and $\mathsf {ct}_{i}^{\mathsf {sum}} = \mathsf {ct}_{i}' + \mathsf {Rot}(\mathsf {ct}_{i}';s)$, where the real parts of first *s* slots in $\mathsf {ct}_{i}^{\mathsf {sum}}$ contains $\vec {y}_{i}$ (see Fig. 7(b)).

From the naive approach, we need *T* number of the matrix-vector multiplication, so that the *CMult* and *Rot* should be computed *T* and 2*T* times, respectively. Here, the implementation cost of *Rot* is larger than *CMult*, so we suggest new packing method to reduce the number of rotation in the whole algorithm. Our main idea is to duplicate and encode *X* multiple times in each ciphertexts, which reduces the number of rotations since the number of summation between the slots in one ciphertexts is reduced.

Now, we propose a new approach for matrix-vector multiplication to reduce the number of constant multiplications and rotations. Our main idea is that we copy each $\vec {x}_{j}$’s several times in encoding step. For the same condition as above example, we can encode the vectors $\vec {x}_{i}$ twice so that we obtain two ciphertexts $\mathsf {ct}_{0} = \mathsf {Enc}(\vec {x}_{0} \| \vec {x}_{0} \| \vec {x}_{1} \| \vec {x}_{1})$ and $\mathsf {ct}_{1} = \mathsf {Enc}(\vec {x}_{2} \| \vec {x}_{2} \| \vec {x}_{3} \| \vec {x}_{3})$. In this case, we define $\tilde {w}_{k, l}$ as in Eq. (2).

2 $$\begin{array}{@{}rcl@{}} \tilde{w}_{k, l} = \left(\vec{w}_{2k}^{(2l)}\left\| \vec{w}_{2k+1}^{(2l)} \right\| \vec{w}_{2k}^{(2l+1)} \left\| \vec{w}_{2k+1}^{(2l+1)}\right.\right) \end{array} $$

for 0≤*k*,*l*<2. Then we get $\mathsf {ct}_{k, l}^{\mathsf {mult}} = \mathsf {ct}_{l} \cdot \tilde {w}_{k, l} = \mathsf {Enc}(\vec {x}_{2l} \odot \vec {w}_{2k}^{(2l)} \| \cdots \| \vec {x}_{2l+1} \odot \vec {w}_{2k+1}^{(2l+1)})$. Thus, by rotating and adding those ciphertexts as $\mathsf {ct}_{k}^{\mathsf {sum}} = \sum _{l=0}^{1} \mathsf {ct}_{k,l}^{\mathsf {mult}}$ and $\mathsf {ct}_{Y, k} = \mathsf {ct}_{k}^{\mathsf {sum}} + \mathsf {Rot}(\mathsf {ct}_{k}^{\mathsf {sum}}; 2s)$, the result ciphertexts contain message vectors as in Eqs. (3) and (4),

3 $$\begin{array}{*{20}l} \mathsf{ct}_{Y, 0} =& \mathsf{Enc}\left(\sum_{j=0}^{3} \vec{x}_{j} \odot \vec{w}_{0}^{(j)} \left\|\sum_{j=0}^{3} \vec{x}_{j} \odot \vec{w}_{1}^{(j)} \right\| \cdots \right), \end{array} $$

4 $$\begin{array}{*{20}l} \mathsf{ct}_{Y, 1} =& \mathsf{Enc}\left(\sum_{j=0}^{3} \vec{x}_{j} \odot \vec{w}_{2}^{(j)} \left\| \sum_{j=0}^{3} \vec{x}_{j} \odot \vec{w}_{3}^{(j)} \right\| \cdots \right), \end{array} $$

which are $\vec {y}_{i}$’s that we desired. In this method, the operations we need are 4 *CMult*s and 2 *Rot*s, while 2 ciphertexts are required for encryption instead of 1 (see Fig. 7(c)).

#### Using imaginary part of message space

The CKKS scheme supports operations between complex numbers, but in applications using real numbers only such as neural networks, the imaginary part is never used. Here, we can make the computation in the encrypted state more efficient by using the imaginary part, which is not used in the plain operation.

In [35], the authors suggested the method to reduce the number of multiplications using CKKS scheme. For four real numbers *a*, *b*, *c*, and *d*, the sum of the two products *ab*+*cd* is equal to the real part of one complex product (*a*+i*b*)(*c*−i*d*), where $\mathsf {i}= \sqrt {-1}$. Since the matrix-vector multiplication we need is also composed of the sum of Hadamard multiplications between vectors, we can reduce the number of ciphertext and the number of constant multiplications by half by combining each vector by two and encoding it into one complex number.

#### Rotate and sum algorithm

For the matrix-vector multiplication, the addition between data packed in one ciphertext is needed. We use *RotSum* algorithm, which repeatedly computes rotation and addition to get summation of desired slots in a ciphertext.

Precisely, we define the algorithm *RotSum*(*ct*,*s*,*t*,*d*) outputs the ciphertext *ct*_*out*_ that is obtained by computing *ct*_*out*_←*ct* and *ct*_*out*_←*ct*_*out*_+*Rot*_*rk*_(*ct*_*out*_;*st*^*j*^) for *j*=0,1,⋯,*d*−1 in order. As a result, if *ct* was an encryption of the message vector $\vec {m} =(m_{i})_{1\le i\le n}$, then *ct*_*out*_ contains the message vector $\vec {m}_{\mathsf {out}} = \left (\sum _{j=0}^{t^{d}-1} m_{[i+js]_{n}}\right)_{1 \le i \le n}$.

#### Putting it all together

Combining all of the above methods, we explain the explicit method used for our implementation. For the rest of this section, we denote the concatenation of vectors by $\|_{i=s}^{t} \vec {a}_{i} = (\vec {a}_{s} \| \vec {a}_{s+1} \| \cdots \|\vec {a}_{t})$ and the repeated concatenation of the same vector by $(\vec {a})^{r} = (\vec {a} \| \vec {a} \| \cdots \| \vec {a})$ (*r* times). Also, we can embed matrices X and W into large matrices with row and column lengths of power-of-2, respectively, so we assume that *s*,*g* are power-of-2’s. Recall that our goal is to compute $\vec {y}_{j} = \sum _{i=1}^{g} \vec {x}_{i} \odot \vec {w}_{j}^{(i)}$ for 0≤*j*<*t* while $\vec {x}_{i}$’s are encrypted, so that the output $\vec {y}_{j}$’s are also encrypted.

As explained before, we copy each $\vec {x}_{i}$’s *m* times when encoding, where *m* is a power-of-2. Since each vector has the size *s* and the number of slots in a ciphertext is *n*, each ciphertext will contain $\ell = 2 \cdot \frac {n}{ms}$ number of different vectors (in both real and imaginary parts). Thus we need total $\frac {g}{\ell } = m \cdot \frac {sg}{2n}$ number of ciphertexts for encoding.

Precisely, we encode $\vec {x}_{i}$’s to each ciphertext *ct*_*i*_ and plain vector $\tilde {w}_{ji}$ by Eqs. (5) and (6) for $0 \le i \le \frac {g}{\ell } - 1$ and $0 \le j \le \lceil \frac {T}{m} \rceil - 1$, respectively. Note that the massage vector in *ct*_*i*_ sequentially contains *m* copies of vector $\vec {x}_{i \cdot \ell + 2q} + \mathsf {i} \cdot \vec {x}_{i \cdot \ell + 2q + 1}$ for each *q*. Here we define $\vec {w}_{jm+r} = \vec {0}$ if *jm*+*r*≥*T*.

5 $$\begin{array}{@{}rcl@{}} \mathsf{ct}_{i} = \mathsf{Enc}\left(\|_{q=0}^{\frac{\ell}{2}-1} \left(\vec{x}_{i \cdot \ell + 2q} + \mathsf{i} \cdot \vec{x}_{i \cdot \ell + 2q + 1}\right)^{m}\right), \end{array} $$

6 $$\begin{array}{@{}rcl@{}} \tilde{w}_{ji} = \|_{q=0}^{\frac{\ell}{2}-1} \left(\|_{r = 0}^{m-1}\left(\vec{w}_{jm + r}^{(i\cdot \ell + 2q)} - \mathsf{i} \cdot \vec{w}_{jm + r}^{(i\cdot \ell + 2q + 1)}\right) \right). \end{array} $$

Next, we compute the multiplication step. For each *j*, we multiply *ct*_*i*_ and $\tilde {w}_{ji}$, add its conjugation and divide it by 2 to get $\mathsf {ct}_{ji}' = \left ((\mathsf {ct}_{i} \cdot \tilde {w}_{ji}) + \overline {(\mathsf {ct}_{i} \cdot \tilde {w}_{ji})}\right) / 2$. Then, by adding them to get $\mathsf {ct}_{j}^{\mathsf {sum}} = \sum _{i=0}^{\frac {g}{\ell }-1} \mathsf {ct}_{ji}'$, we get the ciphertext $\mathsf {ct}_{j}^{\mathsf {sum}}$ which is the encryption of the vector in Eq. (7) for each *j*.

7 $$\begin{array}{@{}rcl@{}} \|_{q=0}^{\frac{\ell}{2}-1} \left(\|_{r = 0}^{m-1} \left(\sum_{i=0}^{\frac{g}{\ell}-1} \sum_{k=0}^{1} \vec{x}_{i \cdot \ell + 2q+k} \odot \vec{w}_{jm + r}^{(i\cdot \ell + 2q+k)}\right) \right). \end{array} $$

Finally, we compute the following summations using *RotSum* so that the first *ms* slots contain the desired sums and are copied to the remaining slots. The Eq. (8) indicates the result of vector operation from Eq. (7) since the summation for (*i*·*ℓ*+*wq*+*k*)’s for all indices *q*,*i*, and *k* works as the summation for all indices from 0 to *q*−1. Hence, the result is the desired vector $\vec {y}_{jm+r}$.

8 $$\begin{array}{@{}rcl@{}} \sum_{q=0}^{\frac{\ell}{2}-1}\sum_{i=0}^{\frac{g}{\ell}-1} \sum_{k=0}^{1} \vec{x}_{i \cdot \ell + 2q+k} \odot \vec{w}_{jm + r}^{(i\cdot \ell + 2q+k)} = \vec{y}_{jm+r}. \end{array} $$

Then for each *j*, we compute the result *ct*_*Y*,*j*_ that contains *j*th group of *m* outputs $\{\vec {y}_{jm + r}\}_{0\le r< m}$ using *RotSum* operation. From Eq. (8), the resulting ciphertext *ct*_*Y*,*j*_ can be computed as Eq. (9).

9 $$\begin{array}{@{}rcl@{}} \mathsf{ct}_{Y, j} &=& \mathsf{RotSum}\left(\sum_{i=0}^{\frac{g}{\ell}-1} \mathsf{ct}_{ji}', ms, 2, \log\frac{\ell}{2}\right)  \\ &=& \mathsf{Enc} \left(\left(\|_{r=0}^{m-1} \vec{y}_{jm + r} \right)^{\frac{\ell}{2}} \right). \end{array} $$

As a result, the desired vectors $\vec {y}_{i} = X \cdot \vec {w}_{i}$ are contained in $\lceil \frac {T}{m} \rceil $ number of ciphertexts $\{\mathsf {ct}_{Y, j}\}_{0\le j < \lceil \frac {T}{m} \rceil }$. Note that the number of rotations used in the Eq. (9) is $\log \frac {\ell }{2} = \log \frac {n}{ms}$ for each *j*, so total $\log \frac {n}{ms} \cdot \lceil \frac {T}{m} \rceil.$

In summary, if we duplicate $\vec {x}_{j}$’s *m* times for encryption, then the required number of rotations is reduced by *m* times, where the number of ciphertexts for encryption in increased by *m* times. Exactly, the number of rotations and multiplications are $\log \frac {n}{ms} \cdot \lceil \frac {T}{m} \rceil $ and $ \frac {sg}{2n} \cdot m \lceil \frac {T}{m} \rceil $, respectively, while the number of ciphertexts for encryption is increased to $m\cdot \frac {sg}{2n}$ (note that the value $\frac {sg}{2n}$ implies the minimum number of ciphertexts to encrypt *X*). Since the number of multiplication does not change asymptotically for *m*, our method provides a time cost trade-off between the number of rotations and encryptions. In our implementation environment, we can reduce the total time cost by using more memory to encrypt data since the rotation cost is the significant part in total matrix-vector multiplication algorithm.

### Approximation of softmax

While softmax layer substantially enhances the performance of the classification, it cannot be directly computed by CKKS scheme since it comprises several non-polynomial operations. Recall that the softmax of a real vector $\vec {v}=(v_{1}, \cdots,v_{t})$ is defined as Eq. (10).

10 $$\begin{array}{@{}rcl@{}} {}\texttt{softmax}(\vec{v}) = \frac{1}{\sum_{i=1}^{t} {\exp(v_{i})}} (\exp(v_{1}), \cdots, \exp(v_{t})) \end{array} $$

Both exponential function and division are not polynomial, so we should replace them by their polynomial approximation. We introduce a proper polynomial approximation technique for each of exponential and division function. In general, in order to approximate a non-polynomial operation by a polynomial, minimax method that minimizes the maximum error value within an interval or Chebyshev approximation that express the function by the series of Chebyshev polynomials are mainly used. However, the minimax approximation loses the increasing property of exponential and the division function, because the sign of the error is not constant. Hence, this method is not suitable in terms of calculating microAUC score. Instead, we suggest an approximation method of the softmax layer that is more suitable for microAUC score. In particular, our approximation method consists of a less number of squaring operations, so it has an advantage to be evaluated over homomorphically encrypted data.

#### Approximation of exponential function

Our approximation method for exponential function comes from the elementary definition of exp(*x*). Precisely, for some *r*, we approximately compute exp(*x*) as Eq. (11).

11 $$\begin{array}{@{}rcl@{}} \exp(x) = {\lim}_{n\to\infty} \left(1+\frac{x}{n}\right)^{n} \approx \left(1+ \frac{x}{2^{r}} \right)^{2^{r}}. \end{array} $$

Note that this formula can be computed by squaring *n* times, so it mitigates the computational overhead accompanied by the HE computation. Also, our approximation of exp(*x*) is monotone on [−2^*r*^,*∞*), so it is more appropriate to approximate softmax functions in terms of getting a high microAUC score compared to other polynomial approximation techniques such as minimax and Chebyshev approximation.

Furthermore, to make the evaluation more stable, we rather consider the approximation of scaled exponential function, AE_*r*,*L*_(*x*) as Eq. (12). From the definition, we expect this funciton to satisfy $ \texttt {AE}_{r,L}(x) \approx \left (\frac {L}{2^{r}}\right)^{-2^{r}} \exp (x). $ If we carefully choose *L* large enough that satisfies $|\frac {2^{r} + x}{L}|<1$ for the all possible choices of *x*, then the values will not rapidly grow during the evaluation. This makes the computation more stable to the HE implementation which usually supports fixed precision bits.

12 $$\begin{array}{@{}rcl@{}} \texttt{AE}_{r,L}(x) := \left(\frac{2^{r} + x}{L} \right)^{2^{r}} \end{array} $$

#### Goldschmidt’s algorithm

Goldschmidt’s divison algorithm [51] is one of the most popular approximation algorithm to compute the inverse of a real number. For *x*∈(0,2), Goldschmidt’s algorithm uses the property as in Eq. (13).

13 $$\begin{array}{@{}rcl@{}} \frac{1}{x} &=& \frac{1}{1-(1-x)} = \sum_{i=0}^{\infty} (1-x)^{i}  \\ &\approx& \sum_{i=0}^{2^{d}-1} (1-x)^{i} = \prod_{i=0}^{d-1} \Big(1+(1-x)^{2^{i}}\Big) \end{array} $$

Note that the approximation converges rapidly as *d* grows, and it uses 2*d*−2 multiplications to evaluate the approximate polynomial of degree 2^*d*^−1. The small number of multiplication gives a great advantage on implementation with HE, because HE multiplication between ciphertext is hugely time consuming. The detailed algorithm is described in Algorithm 3. Here, note that the Algorithm 3 quickly converges to 1/*x* since the ratio between the error and true value is $\frac {a_{d} - 1/x}{1/x} = (1-x)^{2^{d+1}}$.


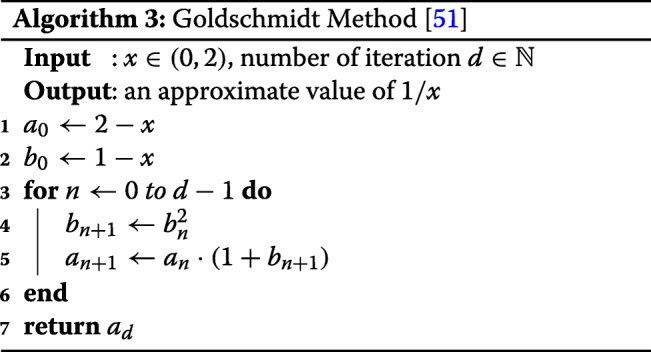


Moreover, we can easily exploit Goldschmidt’s algorithm Gol(·) to approximately evaluate 1/*x* on (0,2*M*), instead of (0,2), by using the equation $ \frac {1}{x} \approx \frac {1}{M} \texttt {Gol} \big (\frac {x}{M}\big). $ We note that the scaling factor *M* should be carefully chosen since the error accompanied by Goldschmidt’s algorithm becomes non-negligiblly large as the input is near 0. Therefore, if we select an overly large *M*, the input values become smaller, resulting in an error that cannot be ignored.

To put approximate exponential function and Goldschmidt’s algorithm together, we now can approximately compute the softmax layer by using HE operations. Denoting $L_{r} = \left (\frac {L}{2^{r}}\right)^{2^{r}}$, we utilize the Eq. (14) to compute approximate softmax function using HE.

14 $$ \begin{aligned} \texttt{softmax}(\vec{v}) &= {\left(\sum_{i=1}^{t} {\frac{\exp(v_{i})}{L_{r}}}\right)^{-1} \left(\frac{\exp(v_{1})}{L_{r}}, \cdots, \frac{\exp(v_{t})}{L_{r}}\right)} \\ &\approx \texttt{Gol}\left(\sum_{i=1}^{t}{\texttt{AE}_{r,L} (v_{i})}\right) \left(\texttt{AE}_{r,L} (v_{1}), \cdots, \texttt{AE}_{r,L} (v_{t})\right). \end{aligned}  $$

The detailed algorithm is described in Algorithm 4.


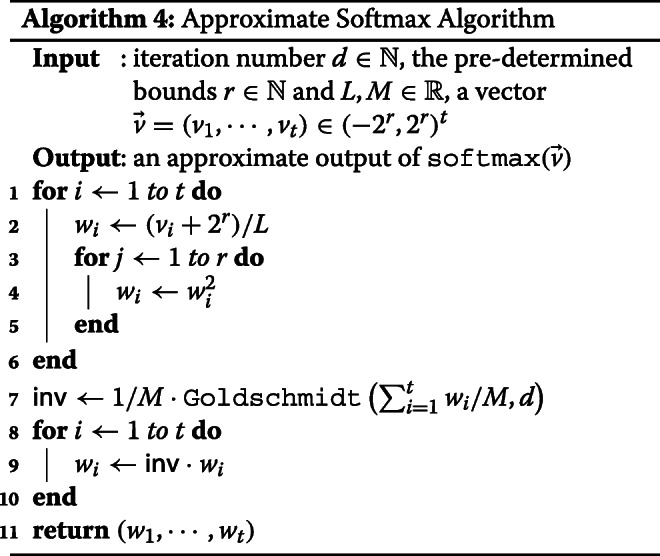


## Data Availability

The dataset is generated and analyzed by the same way as iDASH competition 2020 from the The Cancer Genome Atlas (TCGA) dataset. The dataset is available in [41].

## Abbreviations

HE: Homomorphic encryption
DNN: Deep neural network
CNN: Convolutional neural network
SIMD: Single instrument multiple data
CN: Copy number

## Footnotes

1 which means that an attacker needs more than 2^128^ operations to recover any secret message from a ciphertext with the current best algorithm.
